# Pharmacokinetics of fluticasone furoate, umeclidinium, and vilanterol as a triple therapy in healthy volunteers

**DOI:** 10.5414/CPP54076

**Published:** 2016-01

Erratum to the article “Pharmacokinetics of fluticasone furoate, umeclidinium, and vilanterol as a triple therapy in healthy volunteers” by Noushin Brealey, Ashutosh Gupta, Jessica Renaux, Rashmi Mehta, Ann Allen, and Alex Henderson in *International Journal of Clinical Pharmacology and Therapeutics,* Vol. 53 – No. 9/2015, pp 753-764.

Following an ad hoc review of our paper, we noticed some incorrect data in some of the tables. Upon further investigation it became clear that when Tables 1, 2a and b, 3a and b, 4a and b, and 6 were reproduced for the clinical study report and subsequently copied into our published paper, a number of transcription errors were inadvertently included. An additional QC of the data from the source studies, CTT116415 and 200587, has revealed these were the only errors in the published paper.

We would like to highlight that despite these errors being included in our original report, the conclusions from study CTT116415 and study 200587 remain unaffected and valid as stated in the manuscript. This is because our statistical conclusions and treatment comparisons are based on the 90% confidence interval of treatment ratio estimates (Table 5b on page 759 of the publication), which is not affected by these amends.

We would like to apologize for this unfortunate error.

*Alex Henderson, on behalf of all authors*

The errors have been corrected as follows and highlighted in red/strikethrough (incorrect data) and green (correct data).

**Table 1.** Participant characteristics and demographics.

	Study 1 CTT116415 (n = 44)	Study 2 200587 (n = 44)
Mean age, years (range)	36.5 (20 – 56)	39.1 (19 – 66)
Sex, n (%) Male Female	32 (73) 12 (27)	36 (82) *5 (11)* **8 (18)**
BMI, kg/m^2^ (range)	27.7 (21.1 – 33.3)	N/A
Ethnicity, n (%) Hispanic or Latino Other	4 (9) 40 (91)	*8 (18)* **5 (11)** 39 (89)
Race, n (%) African American/African heritage American Indian/Alaskan Native Native Hawaiian/Other Pacific Islander White (Arabic/North African heritage) White (Caucasian/European heritage)	38 (86) 1 (2) 1 (2) 0 4 (9)	34 (77) 1 (2) 0 1 (2) 8 (18)

BMI = body mass index.

**Table 2a.** FF Plasma PK summary in study 1 (CTT116415).

Parameter	Treatment^a^	N	n	Geometric mean (95% CI)	%CVb
AUC_(0–8)_ (h×pg/mL)	FF/UMEC/VI	43	43	383 (357 – 410)	23.0
	FF/UMEC	43	42	366 (333 – 402)	30.6
	FF/VI	42	42	322 (298 – 348)	25.5
AUC_(0–t)_ (h×pg/mL)	FF/UMEC/VI	43	43	882 (774 – 1,005)	44.3
	FF/UMEC	43	42	765 (653 – 897)	54.4
	FF/VI	42	42	662 (570 – 768)	50.7
C_max_ (pg/mL)	FF/UMEC/VI	43	43	79.4 (71.7 – 88.0)	34.2
	FF/UMEC	43	42	79.2 (71.0 – 88.3)	36.2
	FF/VI	42	42	63.6 (58.0 – 69.6)	29.9
t_1/2_ (h)^*b*^	FF/UMEC/VI	43	29	21.6 *(17.2, 27.1)* **(17.2 – 27.1)**	65.0
	FF/UMEC	43	29	20.7 *(16.7, 25.7)* **(16.7 – 25.7)**	62.2
	FF/VI	42	25	22.4 *(17.6, 28.3)* **(17.6 – 28.3)**	62.5
t_max_ (h)^b^	FF/UMEC/VI	43	43	0.2 (0.1, 2.0)	NA
	FF/UMEC	43	42	0.5 (0.1, 2.0)	NA
	FF/VI	42	42	1.0 (0.1, 2.1)	NA
t_last_ (h)^b^	FF/UMEC/VI	43	43	36.0 (8.0, *48.2* **48.3**)	NA
	FF/UMEC	43	42	36.0 (8.0, 48.2)	NA
	FF/VI	42	42	24.0 (8.0, 48.1)	NA

^a^Total dose as 4 consecutive doses: 400 μg FF, 500 μg UMEC, and 100 μg VI. ^b^Median (min, max). AUC = area under the plasma concentration-time curve; C_max_ = maximum observed plasma concentration; CVb = between subject coefficient of variation; FF = fluticasone furoate; N = total number of subjects who received this study medication, n = number of subjects for whom parameter derived; NA = not applicable; PK = pharmacokinetics; t_1/2_ = terminal phase half-life; t_last_ = time of last measurable concentration; t_max_ = time to C_max_; UMEC = umeclidinium; VI = vilanterol.

**Table 2b.** FF plasma PK summary in study 2 (200587).

Parameter	Treatment^a^	N	n	Geometric mean (95% CI)	%CVb
AUC_(0–4)_ (h×pg/mL)	FF/UMEC(500)/VI	44	44	212 (196 – 229)	26.0
	FF/UMEC(250)/VI	43	43	211 (192 – 232)	31.8
	FF/VI	43	43	219 (200 – 239)	29.6
AUC_(0–t)_ (h×pg/mL)	FF/UMEC(500)/VI	44	44	629 (555 – 713)	43.2
	FF/UMEC(250)/VI	43	43	607 (525 – 702)	49.9
	FF/VI	43	43	644 (566 – 732)	43.8
C_max_ (pg/mL)	FF/UMEC(500)/VI	44	44	*81.4* **81.5** (73.3 – 90.6)	35.9
	FF/UMEC(250)/VI	43	43	81.1 (72.2 – *91.0* **91.1**)	39.1
	FF/VI	43	43	85.7 (76.6 – 95.8)	37.6
t_max_ (h)^b^	FF/UMEC(500)/VI	44	44	*0.2* **0.3** (< 0.1, 2.0)	NA
	FF/UMEC(250)/VI	43	43	*0.2* **0.3** (< 0.1, 2.0)	NA
	FF/VI	43	43	0.2 (< 0.1, 2.0)	NA
t_last_ (h)^b^	FF/UMEC(500)/VI	44	44	24.0 (4.0, 24.1)	NA
	FF/UMEC(250)/VI	43	43	24.0 (*8.0* **6.0**, 24.1)	NA
	FF/VI	43	43	24.0 (8.0, 24.1)	NA

^a^Total dose as 4 consecutive doses: 400 μg FF, 500 μg or 250 μg UMEC, and 100 μg VI. ^b^Median (min, max).

**Table 3a.** UMEC plasma PK summary in study 1 (CTT116415).

Parameter	Treatment^a^	N	n	Geometric mean (95% CI)	%CVb
AUC_(0–4)_ (h×pg/mL)	FF/UMEC/VI	43	43	494 (459 – 531)	23.9
	FF/UMEC	43	42	498 (461 – 537)	24.9
	UMEC/VI	41	41	502 (463 – 544)	26.1
AUC_(0–t)_ (h×pg/mL)	FF/UMEC/VI	43	43	885 (785 – 997)	40.3
	FF/UMEC	43	42	797 (712 – 893)	37.6
	UMEC/VI	41	41	931 (810 – 1,070)	46.2
C_max_ (pg/mL)	FF/UMEC/VI	43	43	1,189 (1,015 – 1,392)	54.9
	FF/UMEC	43	42	1,099 (950 – 1,271)	49.2
	UMEC/VI	41	41	1,217 (1,016 – 1,459)	62.4
t_1/2_ (h)^*b*^	FF/UMEC/VI	43	12	24.6 *(12.0, 50.4)* **(12.0 – 50.4)**	*161* **160.5**
	FF/UMEC	43	19	14.3 *(8.0, 25.4)* **(8.0 – 25.5)**	*179* **178.6**
	UMEC/VI	41	15	17.8 *(8.5, 37.4)* **(8.5 – 37.4)**	*224* **223.8**
t_max_ (h)^b^	FF/UMEC/VI	43	43	0.1 *(0.1, 0.2)* **0.1, 0.3)**	NA
	FF/UMEC	43	42	0.1 *(0.1, 0.2)* **0.1, 0.3)**	NA
	UMEC/VI	41	41	0.1 (0.1, 0.3)	NA
t_last_ (h)^b^	FF/UMEC/VI	43	43	36.0 (8.0, 48.1)	NA
	FF/UMEC	43	42	24.0 (4.0, 48.2)	NA
	UMEC/VI	41	41	36.0 (6.0, 48.3)	NA

^a^Total dose as 4 consecutive doses: 400 μg FF, 500 μg UMEC, and 100 μg VI. ^b^Median (min, max).

**Table 3b.** UMEC plasma PK summary in study 2 (200587).

Parameter	Treatment^a^	N	n	Geometric mean (95% CI)	%CVb
AUC_(0–2)_ (h×pg/mL)	FF/UMEC(500)/VI	44	44	423 (383 – 467)	33.3
	FF/UMEC(250)/VI	43	43	205 (186 – 225)	31.6
	UMEC(250)/VI	43	43	204 (186 – 222)	29.3
AUC_(0–t)_ (h×pg/mL)	FF/UMEC(500)/VI	44	44	770 (688 – 862)	38.4
	FF/UMEC(250)/VI	43	43	323 (282 – 369)	45.6
	UMEC(250)/VI	43	43	310 (275 – 349)	40.4
C_max_ (pg/mL)	FF/UMEC(500)/VI	44	44	1,098 (910 – 1,326)	68.3
	FF/UMEC(250)/VI	43	43	*540* **539** (443 – 657)	71.1
	UMEC(250)/VI	43	43	549 (463 – 651)	59.9
t_1/2_ (h)^*b*^	FF/UMEC(500)/VI	44	15	4.1 *(2.7, 6.2)* **(2.7 – 6.2)**	87.5
	FF/UMEC(250)/VI	43	22	2.3 *(2.0, 2.6)* **(2.0 – 2.6)**	31.6
	UMEC(250)/VI	43	29	2.3 *(2.1, 2.6)* **(2.1 – 2.6)**	27.4
t_max_ (h)^b^	FF/UMEC(500)/VI	44	44	< 0.1 (< 0.1, 0.2)	NA
	FF/UMEC(250)/VI	43	43	**<** 0.1 (< 0.1, 0.1)	NA
	UMEC(250)/VI	43	43	**<** 0.1 (< 0.1, 0.2)	NA

^a^Total dose as 4 consecutive doses: 400 μg FF, 500 μg or 250 μg UMEC, and 100 μg VI. ^b^Median (min, max).

**Table 4a.** VI plasma PK summary in study 1 (CTT116415).

Parameter	Treatment^a^	N	n	Geometric mean (95% CI)	%CVb
AUC_(0–2)_ (h×pg/mL)	FF/UMEC/VI	43	43	307 (289 – 327)	19.9
	FF/VI	42	42	260 (247 – 274)	17.1
	UMEC/VI	41	41	264 (247 – 282)	20.9
AUC_(0–t)_ (h×pg/mL)	FF/UMEC/VI	43	43	522 (482 – 565)	26.4
	FF/VI	42	42	462 (424 – 504)	28.5
	UMEC/VI	41	41	439 (401 – 481)	29.4
C_max_ (pg/mL)	FF/UMEC/VI	43	43	639 (586 – 696)	28.6
	FF/VI	42	42	442 (412 – 474)	22.8
	UMEC/VI	41	41	486 *(445 – 514)* **(445 – 532)**	28.8
t_1/2_ (h)^*b*^	FF/UMEC/VI	43	39	8.6 *(7.4, 10.0)* **(7.4 – 10.0)**	49.7
	FF/VI	42	38	9.0 *(7.4, 10.9)* **(7.4 – 10.9)**	63.8
	UMEC/VI	41	38	7.6 *(6.4, 9.0)* **(6.4 – 9.0)**	53.9
t_max_ (h)^b^	FF/UMEC/VI	43	43	0.1 *(0.1, 0.2)* **(0.1, 0.3)**	NA
	FF/VI	42	42	0.1 (0.1, 0.3)	NA
	UMEC/VI	41	41	0.1 (0.1, 0.3)	NA
t_last_ (h)^b^	FF/UMEC/VI	43	43	*12.0* **12.1** (4.0, 24.1)	NA
	FF/VI	42	42	12.0 (4.0, 24.2)	NA
	UMEC/VI	41	41	12.0 (2.0, 24.0)	NA

^a^Total dose as 4 consecutive doses: 400 μg FF, 500 μg UMEC, and 100 μg VI. ^b^Median (min, max).

**Table 4b.** VI plasma PK summary in study 2 (200587).

Parameter	Treatment^a^	N	n	Geometric mean (95% CI)	%CVb
AUC_(0–6)_ (h×pg/mL)	FF/UMEC(500)/VI	44	44	423 (402 – 445)	17.0
	FF/UMEC(250)/VI	43	43	403 (379 – 428)	20.0
	FF/VI	43	43	408 (384 – 433)	19.8
	UMEC(250)/VI	43	43	371 (347 – 396)	21.9
AUC_(0–t)_ (h×pg/mL)	FF/UMEC(500)/VI	44	44	498 *(449 – 464)* **(464 – 534)**	23.4
	FF/UMEC(250)/VI	43	43	488 *(398 – 467)* **(449 – 531)**	27.5
	FF/VI	43	43	507 *(531 – 534)* **(467 – 551)**	27.3
	UMEC(250)/VI	43	43	436 *(478 – 551)* **(398 – 478)**	30.5
C_max_ (pg/mL)	FF/UMEC(500)/VI	44	44	696 *(580 – 646)* **(646 – 749)**	24.7
	FF/UMEC(250)/VI	43	43	638 *(482 – 549)* **(580 – 701)**	31.4
	FF/VI	43	43	601 *(701 – 749)* **(549 – 658)**	30.1
	UMEC(250)/VI	43	43	529 *(581 – 658)* **(482 – 581)**	30.9
t_1/2_ (h)^*b*^	FF/UMEC(500)/VI	44	19	4.5 *(3.5, 5.7)* **(3.5 – 5.7)**	53.0
	FF/UMEC(250)/VI	43	20	4.8 *(3.6, 6.4)* **(3.6 – 6.4)**	65.9
	FF/VI	43	18	4.9 *(4.0, 5.9)* **(4.0 – 5.9)**	39.7
	UMEC(250)/VI	43	19	4.7 *(3.6, 6.2)* **(3.6 – 6.2)**	61.9
t_max_ (h)^b^	FF/UMEC(500)/VI	44	44	0.1 (0.1, 0.2)	NA
	FF/UMEC(250)/VI	43	43	0.1 (0.1, 0.2)	NA
	FF/VI	43	43	0.1 (0.1, 0.2)	NA
	UMEC(250)/VI	43	43	0.1 (0.1, 0.2)	NA

^a^Total dose as 4 consecutive doses: 400 μg FF, 500 μg or 250 μg UMEC, and 100 μg VI. ^b^Median (min, max).

**Table 6.** Pharmacodynamic assessments in study 1 (CTT116415).

Parameters	Change from baseline in weighted mean (0 – 4 hours)	Treatment comparison (test vs. reference)		
		FF/UMEC/VI vs. UMEC/VI	FF/UMEC/VI vs. FF/VI	FF/UMEC/VI vs. FF/UMEC
Maximum change from baseline in heart rate (bpm)	Test	18.9	18.9	18.9
	Reference	19.1	17.5	5.2
	Difference (95% CI)	–0.2 (–2.6 – 2.1)	1.4 (–0.9 – 3.7)	13.6 (11.3 – 15.9)
Derived heart rate (bpm)	Test	6.18	6.18	6.18
	Reference	6.08	5.97	0.69
	Difference (95% CI)	0.10 (–1.16 – 1.35)	0.20 (–1.05 – 1.46)	5.49 (4.25 – 6.73)
Minimum change from baseline in serum potassium (mmol/L)	Test	–0.30	–0.30	–0.30
	Reference	–0.37	–0.36	–0.24
	Difference (95% CI)	0.06 (–0.01 – 0.14)	0.05 (–0.02 – 0.13)	–0.06 (–0.13 – 0.01)
Derived serum potassium (mmol/L)	Test	–0.123	–0.123	–0.123
	Reference	–0.174	–0.185	–0.062
	Difference (95% CI)	0.051 (–0.022 – 0.124)	0.063 (–0.010 – 0.136)	–0.061 (–0.133 – 0.012)
Maximum change from baseline in blood glucose (mmol/L)	Test	0.544	0.544	0.544
	Reference	0.507	0.452	0.061
	Difference (95% CI)	0.037 (–0.044 – *0.110* **0.117**)	0.092 (0.011 – 0.172)	0.482 (0.403 – 0.562)
Derived blood glucose (mmol/L)	Test	–0.0211	–0.0211	–0.0211
	Reference	–0.0478	–0.0294	–0.1698
	Difference (95% CI)	0.0267 (–0.0354 – 0.0889)	0.0083 (–0.0539 – 0.0705)	0.1486 (0.0871 – 0.2102)

